# Microglia induces Gas1 expression in human brain tumor-initiating cells to reduce tumorigenecity

**DOI:** 10.1038/s41598-018-33306-0

**Published:** 2018-10-16

**Authors:** Susobhan Sarkar, Candice C. Poon, Reza Mirzaei, Khalil S. Rawji, Walter Hader, Pinaki Bose, John Kelly, Jeffrey F. Dunn, V. Wee Yong

**Affiliations:** 1 Department of Clinical Neurosciences, Hotchkiss Brain Institute and the Arnie Charbonneau Cancer Institute University of Calgary, Calgary, Canada; 2 Department of Oncology, Hotchkiss Brain Institute and the Arnie Charbonneau Cancer Institute University of Calgary, Calgary, Canada; 3 Department of Biochemistry and Molecular Biology, Hotchkiss Brain Institute and the Arnie Charbonneau Cancer Institute University of Calgary, Calgary, Canada; 4 Department of Surgery, Hotchkiss Brain Institute and the Arnie Charbonneau Cancer Institute University of Calgary, Calgary, Canada; 5 Department of Radiology, Hotchkiss Brain Institute and the Arnie Charbonneau Cancer Institute University of Calgary, Calgary, Canada

## Abstract

We reported previously that microglia decreased the growth of human brain tumor-initiating cells (BTICs). Through microarray analyses of BTICs exposed *in vitro* to microglia, we found the induction of several genes ascribed to have roles in cell cycle arrest, reduced cell proliferation and differentiation. Herein, we tested the hypothesis that one of these genes, growth arrest specific 1 (Gas1), is a novel growth reduction factor that is induced in BTICs by microglia. We found that microglia increased the expression of Gas1 transcript and protein in glioblastoma patient-derived BTIC lines. Using neurosphere assay we show that RNAi-induced reduction of Gas1 expression in BTICs blunted the microglia-mediated BTIC growth reduction. The role of Gas1 in mediating BTIC growth arrest was further validated using orthotopic brain xenografts in mice. When microglia-induced Gas1-expressing BTIC cells (mGas1-BTICs) were implanted intra-cranially in mice, tumor growth was markedly decreased; this was mirrored in the remarkable increase in survival of mGas1-BT025 and mGas1-BT048 implanted mice, compared to mice implanted with non-microglia-exposed BTIC cells. In conclusion, this study has identified Gas1 as a novel factor and mechanism through which microglia arrest the growth of BTICs for anti-tumor property.

## Introduction

Gliomas are the most common primary brain tumors in adults. The most malignant form, glioblastoma, is refractory to current therapies and is associated with a median survival of only 14.6 months despite surgery and chemoradiotherapy^1^. This dismal prognosis is not only a consequence of the aggressive growth properties of glioblastoma cells, but also their ability to exploit the immune microenvironment^2,3^ that include neutrophils^4^, lymphocytes^5^, microglia and macrophages^3^. Microglia and macrophages are the predominant immune cell type infiltrating gliomas^6,7^, which is not surprising given that they are considered the chief immunoregulatory cells in the brain.

Historically, the recruitment of microglia and macrophages to gliomas was postulated to fend off neoplastic cell establishment and growth given their immunoregulatory functions such as antigen presentation and phagocytosis. While there is evidence that microglia/macrophages may attempt to counteract the glioma cell activity^8–14^, in part through the release of cytotoxic molecules, there is accumulating evidence that microglia and macrophages are immunosuppressed^3,15^ or exploited by gliomas to enhance tumorigenecity^16–20^. Thus, there exists a perpetual “tug-of-war” whereby gliomas attempt to subvert surrounding microglia/macrophages while these innate immune cells endeavor to retain their anti-tumor phenotypes.

A subpopulation of glioma cells known as brain tumor-initiating cells (BTICs) may have particularly significant interactions with tumor infiltrating microglia/macrophages. BTICs are glioma stem cells that play a critical role in the initiation, progression and maintenance of glioma^21–26^. We have shown that as few as ten BTICs deposited into the striatum of NOD-SCID mice can form gliomas^27^. The density of BTICs and infiltrating microglia and macrophages are positively correlated in human tissues^28^. Also, BTICs may be responsible for the mitigation of phagocytosis^15^ and secretion of anti-inflammatory molecules^29^ that mediate immune suppression in the glioma microenvironment. Thus, it is important to further elucidate the relationship between BTICs and microglia/macrophages.

We have previously shown that stimulated microglia-conditioned medium (MCM) reduces the formation of BTIC spheres; in correspondence, genes with ascribed roles in cell cycle arrest, proliferation, and differentiation in BTICs such as *THBS1*, *GADD45B*, *IHNBA*, *BCL6*, *SOD2*^23^ are induced by MCM. In these published microarrays^23^, the expression of growth arrest specific 1 (Gas1), a putative tumor suppressor gene associated with blockade of the G_0_-to-S phase transition^30^, was undetectable across all specimens. However, in a new microarray analysis of BTICs exposed to MCM for 6 h compared to BTIC controls (Supplementary Table 1), we found the additional upregulation of *GAS1* (fold change = 2.9) in MCM-exposed BTICs. As Gas1 affects growth suppression in several cancers^31–33^, and since it has been shown that Gas1 induces cell cycle arrest and apoptosis in conventional glioma cell lines *in vitro* and *in vivo*^34–36^, we tested the novel hypothesis that microglia elevate Gas1 expression in BTICs to reduce their growth.

## Results

### MCM induces Gas1 expression in human BTICs

**Figure 1:**
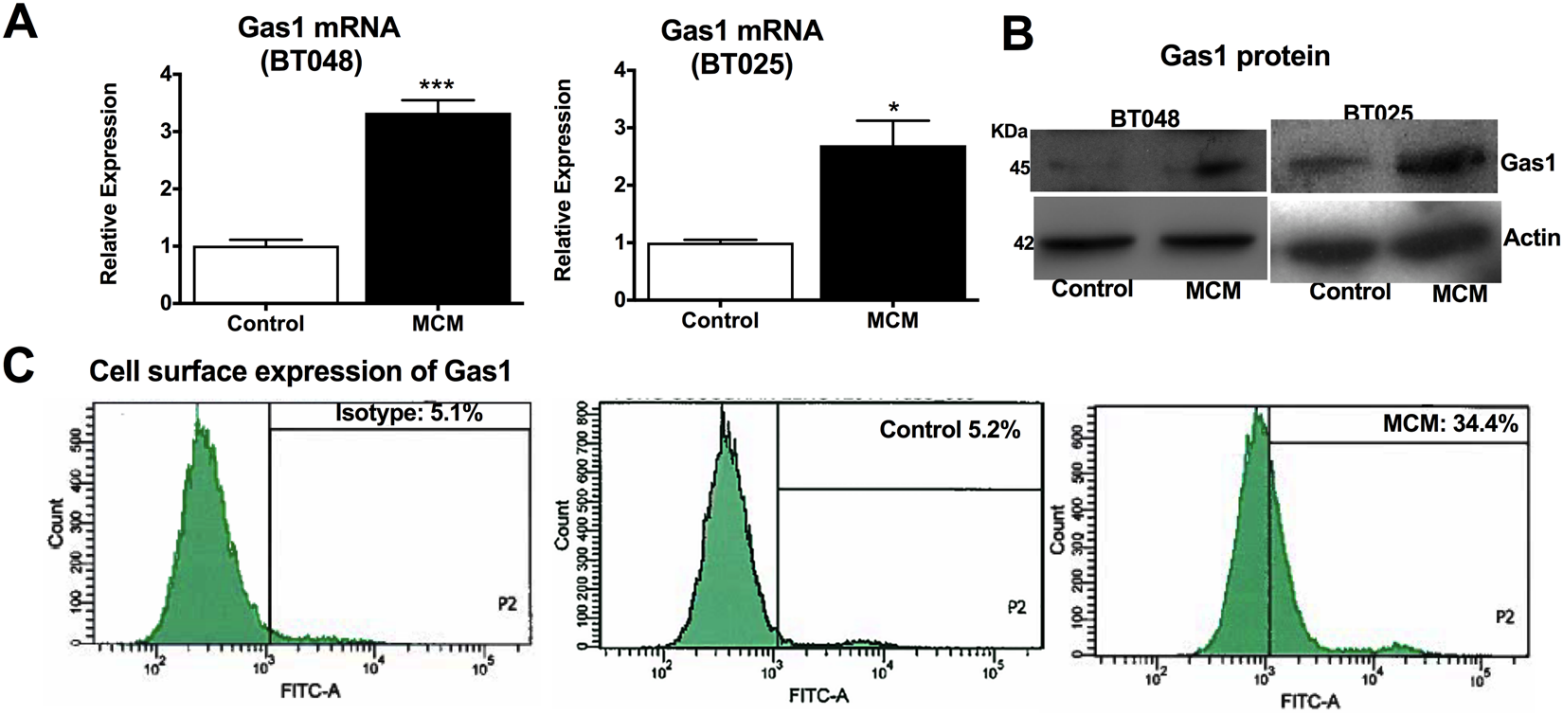
Microglia induces Gas1 expression in BTICs. (**A**) BTICs (BT025 and BT048) exposed to MCM for 6 h showed elevated levels of *GAS1* transcripts normalized to GAPDH, as evaluated using PCR. (**B**) The elevation of Gas1 by MCM was corroborated by Western blot analyses in BTIC cells 6 h after MCM exposure. (**C**) Elevated level of Gas1 expression at the cell surface was also observed through flow cytometry at 6 h after MCM exposure.

We collected conditioned medium from microglia (MCM) that were harvested from patients undergoing brain surgery to treat intractable epilepsy as previously described (23). MCM was then exposed to BTICs for 6 h. Microarray analysis (Supplementary Table 1) identified 703 genes in BT025 cells that were differentially expressed (fold change FC ≥ 1.5) upon MCM exposure compared to control. *GAS1* (FC = 2.9) was amongst the up-regulated genes induced by MCM exposure. We focused on *GAS1* and validated its up-regulation in patient-derived BTIC cells (BT025 and BT048 lines) using qPCR after exposure to MCM for 6 h (Fig. 1A). These findings were corroborated at the protein level with western blots (Fig. 1B). Flow cytometry analysis also demonstrated that MCM increased the cell surface expression of Gas1 after 24 h of exposure in BTICs (34.4%) compared to control (5.2%) (Fig. 1C).

### Attenuation of Gas1 expression in BTICs abolishes the MCM-mediated BTIC growth reduction

**Figure 2:**
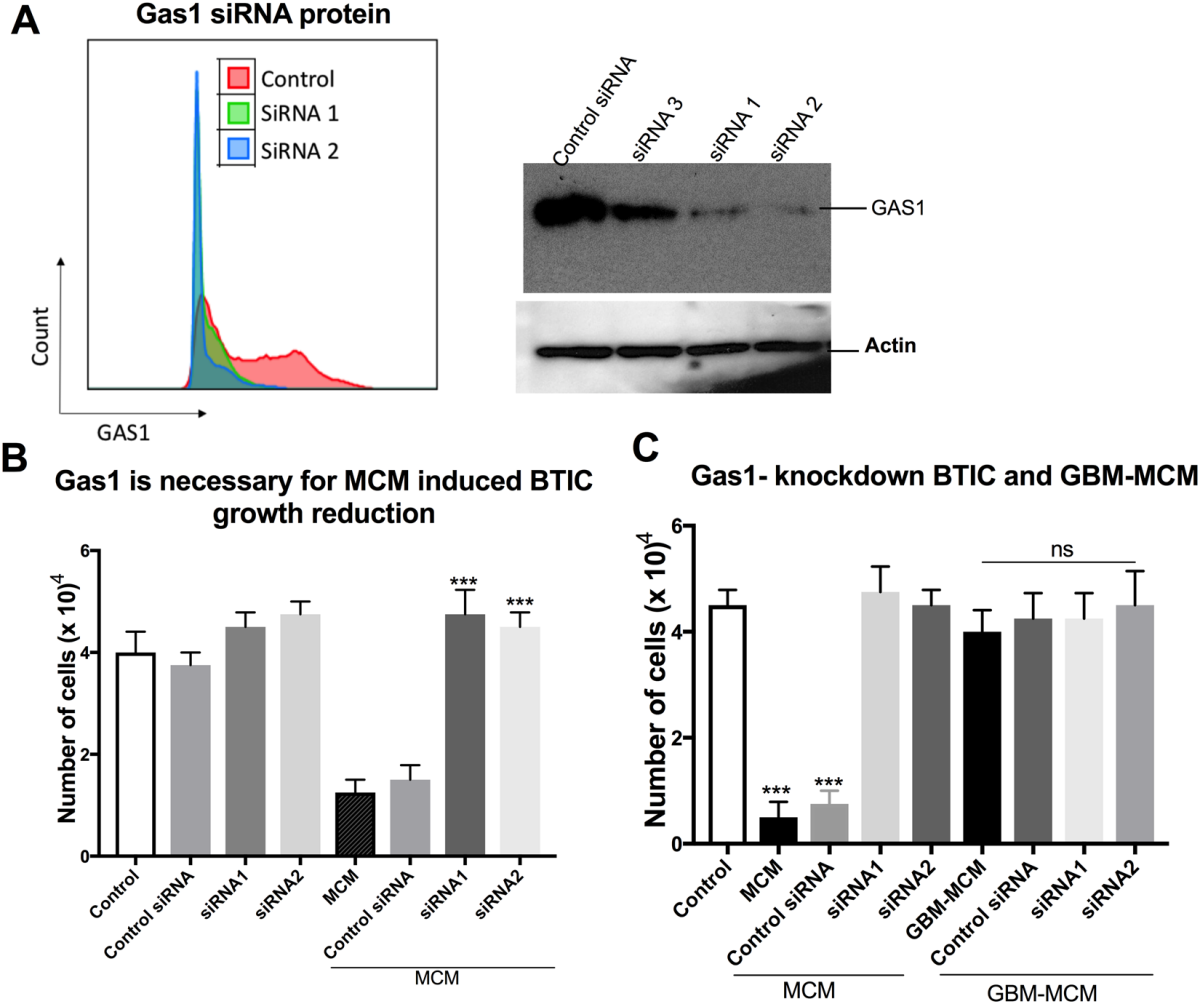
Gas1 is required for microglia-induced BTIC growth reduction. (**A**) The Gas1 protein level in BT048 was decreased by 2 of 3 siRNAs, as evident through western blot analysis and corroborated by FACS. The gels were cut at the relevant molecular weight to highlight the bands of interest. (**B**) Cell counts showed that MCM exposure reduced the number of BT048 cells and this was abrogated in siRNA-treated, Gas1-reduced cultures. In the absence of MCM, the Gas1 targeted siRNAs and control siRNA did not affect BTIC growth (n of 4 where error bar represents sem, ***p < 0.001, with ANOVA comparison to MCM or control siRNA; note that statistical comparisons are only displayed for the MCM conditions). (**C**) Unlike MCM collected from control individuals, GBM-MCM had no effect on Gas1 siRNA-transfected cells. N of 4 where error bars represent sem; ***p < 0.001 compared to all groups.

In order to ascertain the role of Gas1 in microglia-mediated BTIC growth reduction, we used RNAi to knock down *GAS1* expression in BTICs. Figure 2A shows that two siRNAs (siRNA1 and siRNA2) targeting different regions of Gas1 attenuated Gas1 protein expression in BT048 while siRNA3 was relatively ineffective. The reduction of Gas1 was also corroborated by flow cytometry. These transfected cells were then exposed to MCM for 72 h. While MCM potently reduced sphere formation in wild type and control siRNA-transfected BT048, it failed to reduce sphere formation in Gas1 siRNA transfected cells (Fig. 2B). Notably, siRNA1 and siRNA2 transfected cells did not show significant alteration of BTIC growth in absence of MCM exposure at 72 h (Fig. 2B). The effect was also evident in terms of number of BTIC sphere growth (data not shown).

In previous work, we determined that MCM from patients with glioblastoma could not reduce BTIC growth (23), indicating that microglia (and macrophages) in the transformed brain have been subverted to lose their anti-tumor property. Reproducing that dataset, we determined that GBM patient-derived MCM did not reduce BTIC growth (Fig. 2C). In this context, Gas1 knock down BTICs (siRNA1 and siRNA2 transfected cells) were unaffected by GBM patient-derived MCM (Fig. 2C).

### MCM-induced Gas1-expressing BTICs are less tumorigenic in the brain of mice

Based on our observation that microglia induces Gas1 in BTICs, and that MCM has a strong negative impact on BTIC growth in culture, we sought to evaluate the *in vivo* tumorigenicity of BTICs in which expression of Gas1 was induced by exposure to MCM. To test this, MCM-exposed Gas1-expressing BTICs (mGas1-BT025 and mGas1-BT048) were implanted into the right striatum of the female NOD-SCID mice^27^ and compared with MCM-untreated BTIC controls. Control mice and mGas1-BTIC mice were maintained under identical conditions.

**Figure 3:**
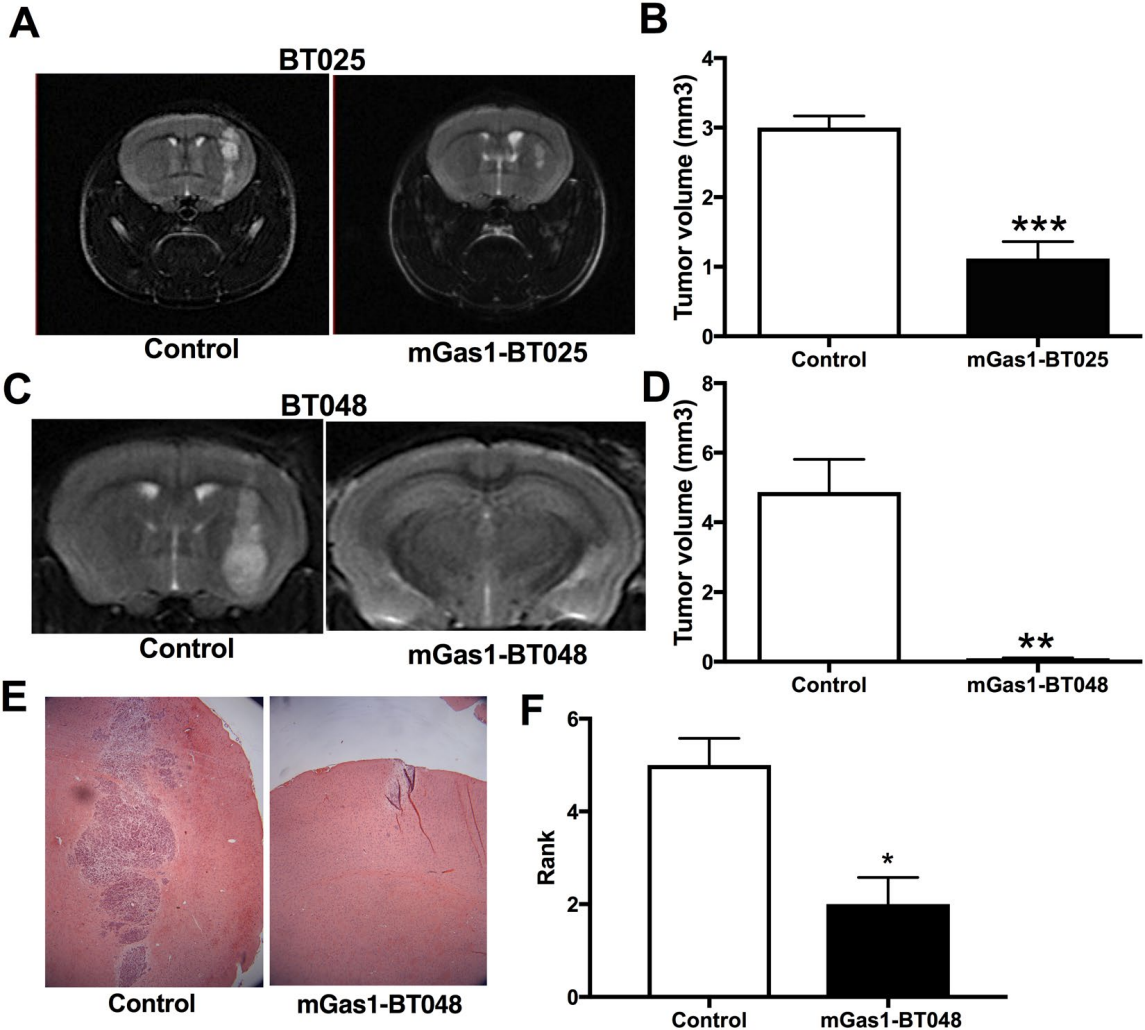
Reduced tumor growth in mice implanted with mGas1-BTICs. (**A**) MRI images at 7 weeks post-implantation (on day 50) showed very large tumor growth in control mice implanted with BT025, whereas in mGas1-BT025 mice the tumor was significantly smaller (quantitation shown in **B**). Similar results were documented for mice implanted with BT048 (MRI performed on day 70; **C**,**D**) that the mice were engrafted with tumor cells was verified by the mice dying at later periods (see Fig. 4B). **p < 0.01, ***p < 0.001 with unpaired t-test (n = 5 for BT025 cells, and n = 4 for BT048 cells; error bar indicates sem). (**E**) In mice killed (n = 3 in each group) immediately after MRI, H&E staining showed massive tumor growth in a representative control (BT048) mouse which was barely evident in a representative mGas1-BT048 animal. Rank order analysis (**F**), where the higher number depicts a larger tumor area. *p < 0.05 (Mann-Whitney U test).

We used T2-weighted MRI to determine the extent of intracranial tumor growth in live animals. The MRIs of mice implanted orthotopically with mGas1-BT025 (Fig. 3A,B) and mGas1-BT048 (Fig. 3C,D) showed a dramatic reduction in tumor mass compared to controls at 7 weeks and 10 weeks, respectively. MRI was performed on day 50 and day 70 for BT025 (Fig. 3A) and BT048 (Fig. 3C) implanted xenografts, respectively. We sacrificed a group of asymptomatic mGas1-BT048 implanted mice at 10 weeks post-implantation and evaluated tumor growth by histology in hematoxylin and eosin (H&E)-stained sections. Figure 3E displays representative H&E-stained sections demonstrating that mGas1-BT048 tumors were notably smaller than those of control BTIC-implanted animals. Thus, consistent with MRI findings, histological analyses corroborated the dramatic reduction of tumor growth for MCM-exposed BTICs compared to BTIC control (Fig. 3F).

### Mice implanted intracranially with mGas1-BTIC implants showed prolonged survival

**Figure 4:**
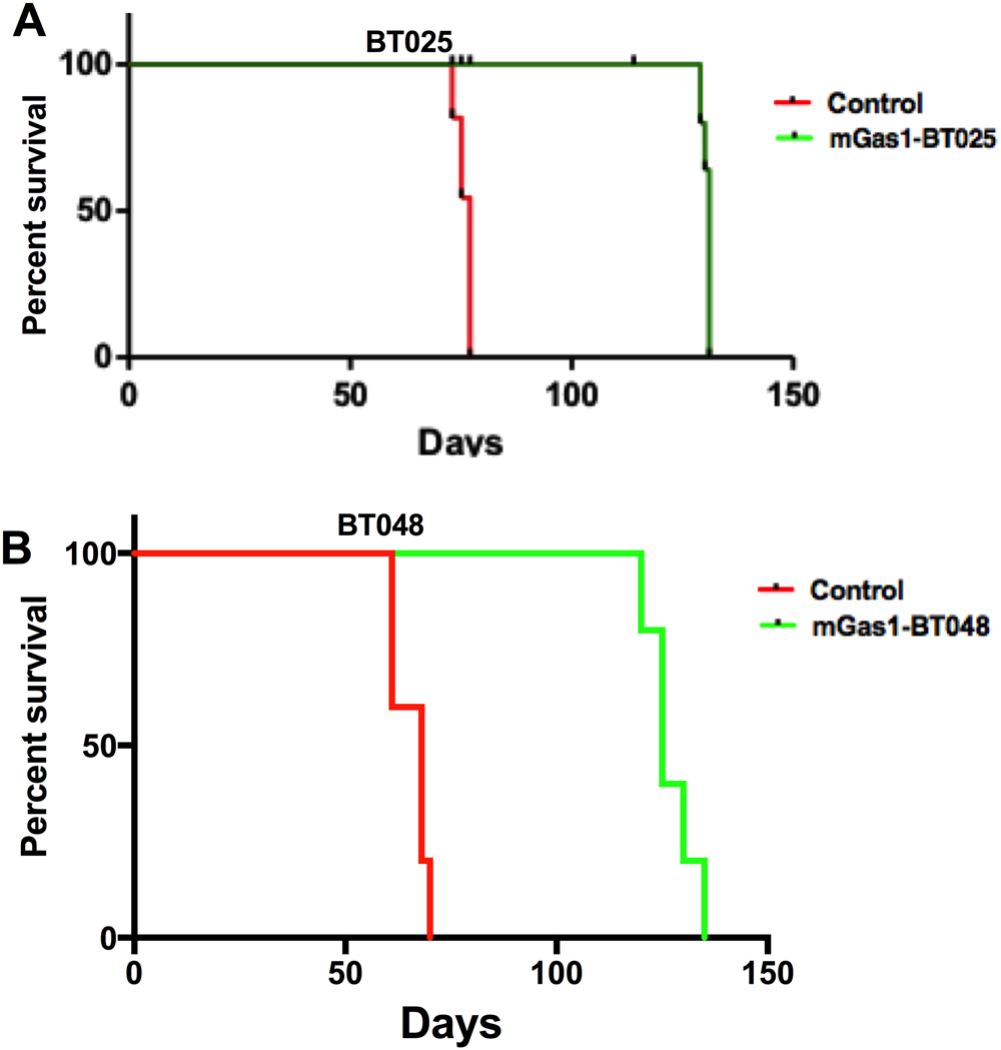
Prolonged survival of mice with mGas1-BTIC implants compared to non-MCM-exposed control BTIC xenografts. Kaplan-Meier analysis showed that the lifespan of mice implanted with MCM-induced Gas1-expressing BTIC implants exceeded that of non-MCM-exposed BTICs for BT025 (**A**, n of 8) and BT048 (**B**, n of 5) lines. Both panels are p < 0.001 (log-rank test).

Mice with intracerebral BTICs eventually die from the expanding tumor mass and the Kaplan-Meier survival curve is a useful tool to evaluate the efficacy of an experimental intervention. Thus, we followed NOD-SCID mice implanted with control BTICs or mGas1-BT025/mGas1-BT048 implants until death ensued. We found that mice implanted with MCM-induced Gas1-expressing BTICs experienced prolonged survival compared to controls harboring intracranial BTICs not exposed to MCM. In animals with BT025 implants, control animals survived 80 days while mGas1-BT025 animals survived up to 130 days (Fig. 4A). Similarly, animals with mGAS1-BT048 implants survived longer (up to 140 days) compared to 70 days for control animals (Fig. 4B).

### GAS1 expression in glioblastoma specimens

**Figure 5:**
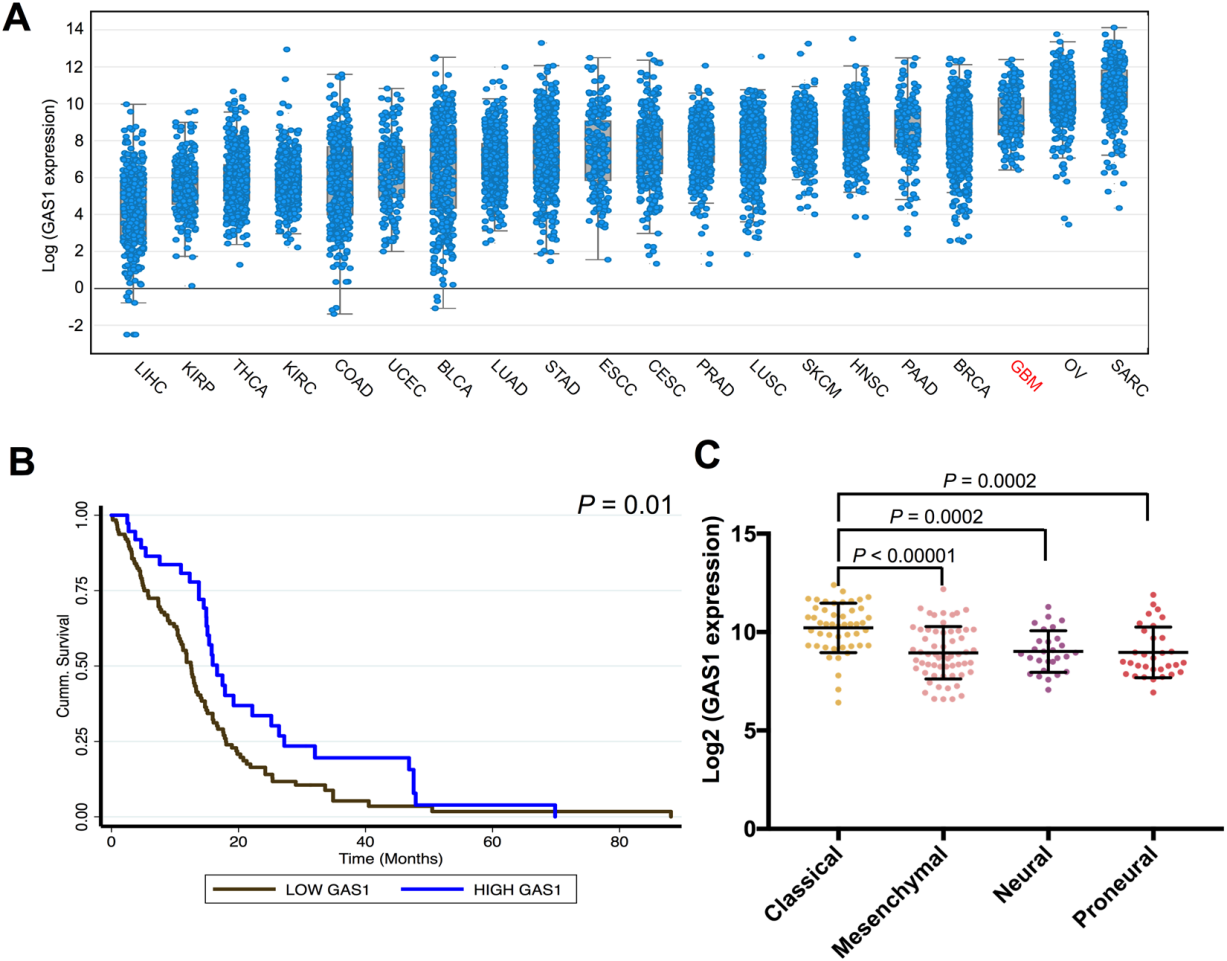
*GAS1* mRNA expression in glioblastoma. (**A**) Results are plotted based on increasing median expression of *GAS1* transcripts across 20 major solid tumour types in TCGA (LIHC: Liver hepatocellular carcinoma; KIRP: Kidney Renal Papillary Cell Carcinoma; THCA: thyroid cancer; KIRC: clear cell renal cell carcinoma; COAD: colorectal adenocarcinoma; UCEC: uterine corpus endometrial carcinoma; BLCA: bladder cancer; LUAD: lung adenocarcinoma; STAD: stomach adenocarcinoma; ESCC: esophageal squamous cell carcinoma; CESC: cervical squamous cell carcinoma; PRAD: prostate adenocarcinoma; LUSC: lung squamous cell carcinoma; SKCM: skin cutaneous melanoma; HNSC: head and neck squamous cell carcinoma. PAAD: pancreatic adenocarcinoma; BRCA: breast cancer; GBM: glioblastoma multiforme; OV: ovarian cancer; SARC: sarcoma. (**B**) Kaplan-Meier curves showing the association between *GAS1* mRNA expression and overall survival in TCGA glioblastoma patients. (**C**) GAS1 mRNA expression in TCGA patients belonging to four glioblastoma subtypes.

To establish the validity of our findings in human glioblastoma samples, we examined *GAS1* mRNA expression across 20 major cancer types in The Cancer Genome Atlas (TCGA), including glioblastoma (GBM; Fig. 5A). *GAS1* was expressed at a very high level in glioblastoma compared to other cancer types and was only lower than *GAS1* levels in ovarian cancers (OV) and sarcoma (SARC). Glioblastoma patients with high *GAS1* expression were associated with significantly improved overall survival compared to those expressing low *GAS1* (logrank *P* = 0.01; Fig. 5B). Finally, we examined the expression of *GAS1* in the four major glioblastoma subtypes (classical, mesenchymal, neural and proneural) in TCGA. *GAS1* expression was significantly higher in the classical subtype compared to the other three subtypes (Wilcoxon ranksum test; Fig. 5C).

## Discussion

Glioblastoma is a notoriously difficult to treat tumor, in part because of treatment resistance imparted by BTICs and also because the immune system is unable to carry out its anti-tumor roles under the influence of these tumor cells. Hence, it is important to develop therapies targeting BTICs and methods to modulate major glioma-associated immune components such as microglia and macrophages to favour a tumor-suppressing phenotype. In this study, we have identified a novel mechanism involving Gas1 through which non-glioma associated microglia curb BTIC growth. This emphasizes the importance of developing therapies that can restore anti-tumor properties in glioma-associated microglia and macrophages.

We have shown that when BTICs were exposed to MCM, Gas1 expression was enhanced significantly at both the transcript and protein levels. We further demonstrated that when Gas1 levels were reduced with siRNA, the anti-proliferative effects of MCM on BTICs was noticeably attenuated, suggesting that Gas1 is a critical mediator of these effects. Several studies have implicated the sonic hedgehog (SHH) pathway in Gas1-mediated tumor growth inhibition^37^. In this context, Gas1 is described as an inhibitor of SHH by sequestering SHH and thereby preventing its binding to Ptc1, thus inhibiting the SHH pathway. On the contrary, Gas1 has also been found to activate SHH signaling. Finally, Gas1 is also known to act as a receptor for Shh, and can activate SHH independent signaling pathway.

To corroborate our *in vitro* results, we evaluated tumor size and survival in an orthotopic immuno-deficient murine BTIC model to maximize translatability to the human condition. We found that MCM-exposed Gas1 expressing BTIC implants led to dramatically smaller tumors and increased survival in both BTIC lines examined. This supports the importance of Gas1 in inhibiting tumor progression and invites the potential of Gas1-modulating agents as anti-glioma treatments. In concordance, glioblastoma patients with higher *GAS1* expression are associated with significantly longer overall survival.

We have previously reported that microglia isolated from resected glioblastoma specimens are deficient in their capacity to reduce the growth of BTICs in culture^23^. It would be of interest to determine whether Gas1 in BTICs is not induced by these glioma-associated microglia and such studies will be conducted in the future. Further, several therapeutics are being tested for their capacity to stimulate microglia and macrophages^38^ and it would be of interest to determine if these stimulated innate immune cells further elevate the expression of Gas1 in BTICs.

In conclusion, our analyses show that Gas1 is an important mediator of BTIC tumorigenesis. Gas1 expression is linked to innate immune system components, microglia and macrophages, which are co-opted by gliomas. This emphasizes the importance of finding innate immune system stimulators since not only will the tumor microenvironment be rendered more hostile to tumor growth, but important downstream pathways such as Gas1 may be activated that can help combat the tumor. Overall, our data suggest that non-tumor-suppressed innate immune cells can exert robust anti-tumor effects on BTICs by inducing Gas1, providing future impetus to search for innate immune cell stimulators that can be used therapeutically to combat gliomas.

## Materials and Methods

### Tissue culture and cells

In previous work, we described the generation of primary BTIC lines from resected specimens of patients with malignant glioblastomas^23,27,39^. Lines BT025 and BT048 were used in the present study, and both were from patients with IDH-wildtype. To propagate the BTIC lines in perpetuity, cells were dissociated and plated into T25 flasks at regular intervals and grown in serum free culture medium supplemented with EGF and FGF (referred to as BTIC medium). Cultures were fed weekly by removing half of the existing medium and replacing with an equal volume of fresh medium. Under such conditions, BTICs formed progressively enlarging spheres.

### Microglia conditioned medium (MCM) and neurosphere assay

Human brain fragments removed during surgical resection to treat drug-intractable epilepsy were used to obtain microglia, as previously described^40^. Cells from 7 epilepsy subjects were used. Informed consent for use of brain material for research was obtained from the patients. The use of these human specimens is approved by the University of Calgary’s Conjoint Health Research Ethics Board; all methods of use of human materials for research were performed in accordance with relevant guidelines and regulations. Fragmented brain tissue was incubated with 0.25% trypsin and 100 μg/ml DNase for 1 h at 37 °C, and dissociated into single cells by forcing them through a mesh of 130 μm pore size. Following centrifugation through 30% Percoll, which allowed myelin, debris and red blood cells to be separated from viable neural cells, the latter cell layer was removed, centrifuged, washed, and plated into T-25 uncoated flasks. Feeding medium was 10% fetal bovine serum in minimum essential medium supplemented with 0.5% dextrose. Microglia from adult human brain are very adherent cells, as opposed to other cell types (e.g. oligodendrocytes), on uncoated flasks. Thus, one day after plating, floating cells were removed to leave behind adherent microglia of over 95% purity. The latter was removed by retrypsinization 2–3 days after, and 2 × 10^6^ microglia were replated into an uncoated well of a 6-well plate and incubated for 48 h with 1 ml of BTIC medium supplemented with EGF and FGF; cells were kept in a 5% CO_2_ incubator at 37 °C during this time. The conditioned medium subsequently collected was referred to as MCM. When the conditioned medium was from glioblastoma-derived microglia, it was noted as GBM-MCM.

To study the response of BTICs to MCM, freshly dissociated BT025 or BT048 cells were plated at a density of 10,000 cells/100 µl in control (non-cell exposed) BTIC medium or MCM. Cultures were maintained at 37 °C in a 5% CO_2_ incubator. After allowing the cells to form spheres for 72 h, four random fields per well were photographed under a 10X objective with a phase contrast microscope, and the number of spheres over 60 μm in diameter was tabulated as previously described^23^.

### Microarray analyses

To determine the effects of MCM on BTIC gene expression, BT025 cells were exposed to MCM for 6 h and subjected to microarray analysis as described elsewhere^22,23^.

### Quantitative real-time PCR (qPCR) for Gas1

BT025 or BT048 cells were lysed in 300 µl TRIzol reagent (Invitrogen, Carlsbad, CA, USA) by leaving plates at room temperature for 5 min before the content of the well was harvested and stored at −80 °C prior to use. Following extraction (Qiagen, Mississauga, Canada), RNA was first treated with DNase (Promega, Madison, WI, USA) and reverse transcribed using Superscript II reverse transcriptase (Invitrogen, Carlsbad, CA, USA). The resulting cDNA was used as a template for the BioRad iCycler MyiQ detection system and 2x SYBR green mastermix (Qiagen, Mississauga, Canada). Every primer that was used was purchased from Qiagen. Expression of gene transcripts was normalized against GAPDH. Relative expression levels for genes of interest was determined using the formula 2 − ΔCT where ΔCT = CT (gene of interest) − CT (housekeeping gene).

### Western blot analyses and flow cytometry analysis of Gas1 expression

Cell lysates were prepared as described before^41^. Equal amounts of protein were electrophoresed in 10% SDS-PAGE under reducing conditions, and transferred to a PDVF membrane (Millipore). The latter was blocked with 10% milk in saline overnight, and was then probed for 24 h with rabbit anti-Gas1, C-terminal (SAB4501120, SIGMA). A secondary antibody anti-rabbit HRP (1:10,000) was added for 1 h, and bands were detected using the enhanced chemiluminescence detection kit (Amershm Bioscience, Piscataway,NJ) and a gel documentation system. Gas1 expression was also carried out using flow cytometry with the same rabbit anti-Gas1, C-terminal antibody.

### Knock down of Gas1 gene in BTICs using RNAi

Three pre-designed siRNAs (21 oligonucleotides in length) were used to target human Gas1. The annealed siRNAs were analyzed by non-denaturing PAGE. A negative control siRNA, composed of a 19-bp scrambled sequence with 3 deoxythymidine overhangs, was used. The sequences have no significant homology to any known gene sequences from mouse, rat, or human databases. For transfection with siRNAs, BTICs were plated in 12-well plates and were incubated with lipofectamine (Invitrogen). After 24 h, cells were harvested for flow cytometry and neurosphere assay described above.

### Animal implantation of mGas1-BT025 and mGas1-BT048 cells

BTIC lines BT025 and BT048 were exposed to MCM and confirmed for Gas1 expression prior to implantation into the brains of NOD-SCID mice. Hence they are denoted as mGas1-BT025 and mGas1-BT048 cells. BTIC spheres were then dissociated to produce single cell suspensions for implantation. Ten thousand viable cells were resuspended in 2 µl of phosphate-buffered saline and implanted into the right striatum of 6–8 week old female NOD-SCID mice (Charles River, Montreal) stereotactically^27^. Animals were returned to their cages and allowed free access to food and water. Animals were weighed every other day and observed for neurological symptoms. The use of mice for these experiments is approved by the Animal Care Committee of the University of Calgary and in concordance with guidelines of the Canadian Council on Animal Care.

### MRI evaluation of tumor growth and survival

Between 7 to 10 weeks post-implantation, an MRI of the brain was conducted at the University of Calgary Experimental Imaging Center as previously described^27^ using a 9.4 T Brunker horizontal-bore MR system. Images were analyzed using Marevici software.

We also performed survival analysis with Kaplan–Meier curves. Survival endpoints complied with protocols approved by the Animal Care Committee at the University of Calgary in accordance with research guidelines from the Canadian Council for Animal Care. They were defined as mice that were moribund (negligible limb movement and loss of 25% of body weight compared to the preceding few days) or mice that were found deceased in their cages. Upon sacrifice, brains were removed and immersed in 10% buffered formalin, paraffin-embedded and sectioned for histology.

### Histological analysis of intracranial tumors

In order to assess tumor size in asymptomatic mice, animals were sacrificed ten weeks after implantation. The whole brain was removed, cut into blocks, fixed in 10% buffered formalin and embedded in paraffin. Six μm sections were taken every 120 μm through the entire brain. The sections were deparaffinized, rehydrated and stained with hematoxylin and eosin (H&E). For rank order analysis of the size of tumor across mice, the brain section with the largest tumor area of each mouse was subjected to blinded ranking of one image against another. This was done for all the images. At the end of the ranking, the scores for each image are added up and the highest number is ascribed to the largest tumor area.

### Bioinformatic analyses

Visualization of *GAS1* expression in The Cancer Genome Atlas (TCGA) cancer types was performed on cBioPortal (http://www.cbioportal.org/) using RNAseq data. RNAseq (level 3) data was downloaded from the Broad Institute Firehose GDAC (https://gdac.broadinstitute.org/). *GAS1* mRNA expression in glioblastoma subtypes was quantified using the STATA 15 statistical software package (College Station, TX), Wilcoxon ranksum test was used to assess statistical significance. The association between *GAS1* mRNA expression and survival in glioblastoma was visualized using Kaplan Meier curves.

### Statistical analysis

Kaplan-Meier survival curves were analyzed for statistical difference between groups using the log-rank test. For analysis of differences in sphere formation in culture, the one-way ANOVA with posthoc Tukey’s comparisons was used for multiple groups, while the t-test was used for groups of two specimens. For the rank order analysis of the size of tumor across mice, the unpaired Mann-Whitney U test was used.

## Electronic supplementary material

Supplementary Table 1
